# Novel Collagen-Polyphenols-Loaded Silica Composites for Topical Application

**DOI:** 10.3390/pharmaceutics15020312

**Published:** 2023-01-17

**Authors:** Mihaela Deaconu, Ana-Maria Prelipcean, Ana-Maria Brezoiu, Raul-Augustin Mitran, Gabriela Isopencu, Cristian Matei, Daniela Berger

**Affiliations:** 1 CAMPUS Research Institute, University “Politehnica” of Bucharest, 313 Splaiul Independentei, 060042 Bucharest, Romania; 2 Faculty of Chemical Engineering and Biotechnologies, University “Politehnica” of Bucharest, 1-7 Polizu Street, 011061 Bucharest, Romania; 3 National Institute of R&D for Biological Sciences, 296 Splaiul Independetei, 060031 Bucharest, Romania; 4 “Ilie Murgulescu” Institute of Physical Chemistry, Romanian Academy, 202 Splaiul Independentei, 060021 Bucharest, Romania

**Keywords:** mesoporous silica, polyphenols, collagen, natural compounds, porous scaffold

## Abstract

Lesions can affect skin functions and cause a simple issue, such as dehydration, or more challenging complications, such as bacterial infections. The purpose of this study was to design composites for topical application that can prevent and/or assist in bacterial infections and support cell regeneration using natural components. A polyphenolic extract obtained from *Salvia officinalis* was embedded in functionalized mesoporous silica nanoparticles for better stability, followed by their distribution into a collagen porous scaffold. The resulting polyphenols-loaded MSN exhibited enhanced antibacterial activity and good cytocompatibility. Improved thermal stability of the collagen porous scaffold was obtained due to the presence of the functionalized MSN. For the first time, collagen-polyphenols-loaded silica composites were reported in the literature as potential wound dressings. The newly developed composites showed excellent sterility.

## 1. Introduction

The skin is the largest human organ, and, in case of injury, it is the first and most affected. It has three main functions: protection, thermoregulation, and sensation. Lesions caused to the skin affect these functions and can lead to multiple complications, such as bacterial infections, hypothermia, impairment of the immune system, dehydration, and wounds that, in the long-term, turn into scars [1]. Among skin lesions, burn wounds represent one of the most complex and traumatizing injuries associated with high mortality rates, causing not only physical impairment for the patient but also mental distress [2]. The World Health Organization estimates that, every year, 180,000 burn injuries have a fatal outcome, most of them occurring in low- and middle-income countries [3].

Collagen is used in wound dressing applications in various forms depending on the manufacturing technique: porous scaffolds, electrospun matrices, films, hydrogels, or microspheres [4]. The interconnected pore array of porous scaffolds allows cells access into the scaffold and ensures their adhesion to the collagen sheets promoting cell proliferation [5]. Furthermore, the porous structure enables bidirectional transport: (i) oxygen passes through the scaffold toward the epithelial tissue, preventing hypoxia and thus enhancing the healing process, and (ii) the exudates and metabolic wastes are absorbed from the wound [6]. Marine sources can be used to obtain collagen and its derivatives, such as gelatin or peptides [7]. Collagen obtained from marine sources is considered a better alternative to mammalian ones due to the high risks associated with the latter, such as initiation of immune reactions or transmission of zoonotic diseases (e.g., spongiform encephalopathy of bovines [8]). The collagen source for the composites designed in this paper is *Rapana venosa*, a predatory and invasive sea snail harvested from the Black Sea.

Mesoporous silica nanoparticles (MSN) are ideal candidates for adsorption and transport of biologically important molecules due to their porous structure, having high specific surface area and total pore volume, which are critical parameters in biological molecules adsorption [9]. The surface properties of MSN can be easily tailored through functionalization. The silanol groups on the MSN surface can undergo condensation reactions in post-synthesis functionalization. This is an important feature that ensures electrostatic interactions between the silica surface and guest molecules, tuning the release profiles of the biologically active compounds based on interactions between the functional groups of silica nanoparticles and cargo molecules [10,11]. The degradation product of MSN is orthosilicic acid, which is non-toxic, being excreted through urine [12]. This is the main reason why silica is biocompatible, and the Food and Drug Administration has ‘generally recognized as safe’ use of silica as a food additive [13]. In fact, orthosilicic acid has been known to be involved in biological processes such as bone mineralization, collagen synthesis, or immune system stimulation [14]. There are many reports on use of mesoporous silica for bacterial infections as a strategy to combat drug resistance [15]. This is a continuously developing research topic that considers use of conventional antibiotics in innovative drug delivery systems [9] or antibiotic-free strategies that are based on the intrinsic antibacterial features of nanoparticles or natural compounds [16].

Due to misuse of antibiotics, researchers are looking for new strategies to reduce antimicrobial resistance [17]. In addition to the usual antibacterial agents (i.e., antibiotics), natural products, such as polyphenolic extracts, are gaining interest for biomedical applications [18]. Polyphenolic extracts are known for their multiple biological activities, such as antioxidant, antidiabetic, antimicrobial, and/or antifungal activities, as well as a synergistic effect in cancer therapy [19,20,21].

Complications arising after skin lesions call for fast intervention and developing complex composites that can address issues, such as bacterial infections or skin regeneration. Composites comprising collagen and inorganic materials have previously been reported for biomedical applications in tissue engineering. For bone tissue engineering, composites often integrate hydroxyapatite [22], while most mesoporous silica composites are designed for epithelial tissue engineering [23,24,25,26,27,28]. Most collagen–silica composites were designed as hydrogels [23,24,25,26], and a few papers mentioned development of porous scaffolds [27,28,29]. Composites designed for epithelial tissue engineering include antibiotics to enhance their antibacterial features [24,30], but alternative collagen composites based on polyphenols have also been proposed [31,32]. Perumal R.K. et al. reported silicified collagen porous scaffolds with grass extract for tissue engineering applications [33].

Our study introduces new collagen-polyphenols-loaded silica composites for topical application with multiple functionalities, such as prevention and/or assisting infection, using natural resources: collagen extracted from *Rapana venosa* and polyphenols obtained from *Salvia officinalis* (common sage). In this study, a polyphenolic extract prepared from *Salvia officinalis* was utilized because it exhibited good antibacterial activity against both Gram-positive and Gram-negative strains, even higher than the used positive control, gentamicin [34]. Moreover, common sage extract has been shown to have good cytocompatibility and antiproliferative activity against healthy cells and various malignant cells, respectively [35], which recommends it as a good option for treatment of skin lesions in any medical condition.

## 2. Materials and Methods

### 2.1. Materials

Tetraethyl orthosilicate (TEOS, Fluka, Seelzer, Germany), triethanolamine (TEA, Sigma-Aldrich Co., Merck Group, Darmstadt, Germany), cetyltrimethylammonium chloride (CTAC, 25 %wt in water, Sigma-Aldrich Co.), poly(ethylene glycol) methyl ether (PEG, average M_n_ 550, Sigma-Aldrich), (3-aminopropyl)triethoxysilane (APTES, 99% Sigma-Aldrich), 3-(triethoxysilyl)propionitrile (CETES, 97%, Sigma-Aldrich), ammonium chloride (Sigma-Aldrich), toluene (Riedel de Haen, Honeywell Riedel-de Haën, Seelzer, Germany), acetone (Sigma, Merck Group, Darmstadt, Germany), absolute ethanol (Sigma-Aldrich), 36.5–38% hydrochloric acid, sulfuric acid 98% (Merck), acetic acid (>99.7%, Sigma-Aldrich Co.), Folin–Ciocalteu (Sigma-Aldrich), 2,2′–azino–bis(3–ethylbenzothiazoline–6–sulphonic acid) (ABTS, Sigma-Aldrich Co.), and potassium persulphate (K_2_S_2_O_8_, Sigma-Aldrich Co.) were used as received. Ultrapure water (Millipore Direct-Q3 UV water system, version Q3 UV, product no. C9185, Merck Group, Darmstadt, Germany, equipped with a Biopack UF cartridge) was used for all experiments. Collagen was provided by the National Institute of Research and Development for Biological Sciences, Bucharest, Romania and prepared as previously described [36].

### 2.2. Mesoporous Silica Carriers Synthesis

Pristine mesoporous silica nanoparticles (MSN) were prepared through a newly reported sol–gel synthesis. Tetraethyl orthosilicate (TEOS) and triethanolamine (TEA) were first heated at 90 °C for 20 min, under static conditions, and then 3.5% CTAC aqueous solution was added to the mixture under vigorous magnetic stirring. Last, a PEG aqueous solution (50 mg/mL) was added dropwise, and the reaction mixture was heated at 100 °C overnight. The TEOS: TEA: CTAC: PEG: H_2_O molar ratio was 1: 0.2: 10.4: 0.05: 130. The structure-directing agent was removed first by extraction in an ethanolic ammonium chloride solution, followed by calcination at 550 °C for 5 h.

The functionalized mesoporous silica nanoparticles were prepared by grafting organic functional groups on pristine MSN through the post-synthesis approach using MSN: organosilane molar ratio of 1: 0.2. The sample denoted *MSN–NH_2_* was prepared using (3-aminopropyl)triethoxysilane and the method previously described [11]. The *MSN–COOH* support was obtained by condensation reaction between 3-(triethoxysilyl)propionitrile and silanol groups of pristine MSN, followed by hydrolysis of the cyanide groups to carboxylic ones in 50% wt of aqueous sulfuric acid solution [11].

### 2.3. Polyphenols-Loaded MSN Preparation

Polyphenolic extract was obtained from *Salvia officinalis* (common sage) by ultrasound-assisted extraction (Bandelin Sonorex Digitec ultrasonic bath—Berlin, Germany) at 40 °C, in absolute ethanol, at a vegetal material/solvent ratio of 1/30 (*w*/*v*). The extraction was carried out in three 15 min. steps, with solvent replacement in each stage. The extract was evaporated using a rotary evaporator (RE100-Pro, DLAB Scientific, Beijing, China) until it reached a constant mass and then was redissolved at a desired concentration. After preparation, *Salvia officinalis* polyphenolic extract (denoted *S*) was adsorbed into functionalized MSN mesopores through impregnation, followed by solvent evaporation under vacuum. First, the mesoporous carriers were dried under vacuum, at room temperature, for 12 h, and then the corresponding volume of polyphenolic extract (15 mg/mL) was added to the support to obtain composites with a theoretical content of 25 %wt extract. The polyphenolic-extract-loaded MSN were denoted *S@MSN-*x, where x = NH_2_ or COOH.

### 2.4. Radical Scavenger Activity Testing

Radical scavenger activity (RSA) of polyphenols-loaded MSN was assessed against 2,2′-azino-bis(3-ethylbenzothiazoline-6-sulfonic acid)diammonium salt (ABTS) according to the method developed for solid samples. The detailed method is presented elsewhere [37]. In summary, 2.95 mL ABTS˙⁺ solution was added to 50 µL free polyphenolic extract, to the corresponding amount of polyphenols-loaded MSN, or to the corresponding amount of silica carriers. The experiment was carried out in duplicate under dark conditions in glass vials under continuous shaking using an orbital shaker (Grant bio-PSU-10i, Grant Instruments, Cambridge, United Kingdom) for 6 h. The solids were separated, and the absorbance of the solution was measured at 753 nm (Shimadzu UV-1800, Shimadzu Corporation, Kyoto, Japan). For the free extract, results were presented as Trolox equivalents (TE). For the embedded extracts, RSA was computed as follows: RSA (%) = [(A_ABTS_ − A_sample_)/A_ABTS_] × 100, where *A_ABTS_* is the absorbance of ABTS radical solution and *A_sample_* is the absorbance of embedded extract–ABTS mixture. Aqueous ABTS˙⁺ solution was used as control.

### 2.5. Antimicrobial Activity Testing

Antimicrobial activity was tested against both Gram-positive and Gram-negative bacterial strains, *P. aeruginosa* (ATCC 27853) and *S. aureus* (ATCC 25923), respectively. Quantitative analysis of the antibacterial potential of the extract and encapsulated extracts was carried out by using the method of serial microdilutions in Mueller–Hinton broth in 96-well plates. The aqueous suspensions were serially 2-fold diluted; negative control was represented by culture medium, while positive one by bacterial cultures. Plates containing fresh standardized bacterial inoculum and the tested extracts were incubated at 37 °C for 24 h. The minimum inhibitory concentration (MIC) was established macroscopically as the last concentration at which the turbidity was observed, and it was further confirmed spectrophotometrically. The minimum bactericidal concentration (MBC) was established by further cultivating on Mueller–Hinton agar the samples incubated with the established MIC for each tested sample. The concentration that managed to kill 99.9% of the bacterial cells after 24 h was confirmed as MBC. Experiments were performed in duplicate.

### 2.6. Cytocompatibility Testing

The biological evaluation of samples was performed in agreement with the law for ethics in research 206/2004, completed with OUG 28/2011, harmonized with European specifications in force. Standardized cell lines were used for the in vitro experiments by following the manufacturer guidelines. No primary cell cultures or animal studies were used in the experimental set-up.

Human normal keratinocytes (HaCat) were cultivated in RPMI supplemented with 10% FBS and 1% PSN antibiotic mixture at 37 °C and 5% CO_2_. For the cytocompatibility assay, the cells were seeded in 96-well culture plates at a density of 5 × 10^4^ cells/mL and incubated in standard conditions to allow cell adhesion. After 24 h, the culture medium was replaced with fresh medium and the tested aqueous suspensions at various concentrations, 5, 30, 50, and 100 µg/mL. The evaluation was carried out at 24 h and 72 h, and the control was considered the untreated cell culture. Results were expressed as mean value ± standard deviation (n = 3). Statistical analysis of the data was performed using two-tailed, two-sample equal variance Student’s *t*-test (Microsoft Office 365 Excel software) on each pair of interest.

### 2.7. Evaluation of Antiproliferative Activity

#### 2.7.1. Cytotoxic Effect

Human Mel-Juso skin carcinoma cells were cultivated in RPMI supplemented with 10% FBS and 1% PSN antibiotic mixture at 37 °C and 5% CO_2_. To determine the cytotoxic potential, cells were seeded and treated under the same conditions as the normal human keratinocytes, and the MTT test was performed at the same time points.

#### 2.7.2. Apoptotic Effect

Flow cytometry analysis was conducted as previously described [38]. Mel-Juso cells were seeded in 12-well culture plates, allowed to attach for 24 h, and then treated with the indicated concentrations of samples for 72 h at 37 °C. Cells were trypsinized, washed with phosphate buffered saline (PBS), and collected by centrifugation at 1500 rpm for 5 min at room temperature. Apoptosis was analyzed using Dead Cell Apoptosis Kit. Aliquots of cells (1 × 10^5^ cells) were double stained with fluorescein-isothiocyanate-labeled Annexin-V and propidium iodide for 15 min at room temperature in the dark according to the manufacturer’s instructions (Invitrogen). Immediately after staining, samples were analyzed on a LSR II BD flow cytometer (Beckton Dickinson, San Jose, CA, USA).

### 2.8. Preparation of Collagen Porous Scaffold Composites Preparation

Collagen porous scaffolds (*C*) were prepared by freeze-drying the collagen solution. Thus, pristine collagen porous scaffolds were prepared from 0.5 mL collagen acidified (0.1 M acetic acid) solution (1.06 mg/mL) and 0.5 mL water. Samples were lyophilized at −40 °C for 24 h (Martin Christ Alpha 2–4 LSCbasic, Martin Christ Gefriertrocknungsanlagen GmbH, Osterode am Harz, Germany).

The collagen porous scaffold-polyphenols-loaded silica composites were prepared from 0.5 mL collagen acidified solution (1.06 mg/mL) and 0.5 mL S@MSN-x suspension in water. Thus, common sage extract-loaded functionalized MSN were dispersed in water assisted by ultrasounds and then the suspension was added to the collagen solution. Before lyophilization, the mixture was placed on an orbital shaker for 1 h to allow a good dispersion of the S@MSN-x (10 or 20 mg/mL) into the collagen solution.

### 2.9. Sterility Testing

Sterility testing of collagen composites was performed by direct inoculation, as described in the European Pharmacopoeia [39]. Thus, collagen porous scaffolds and collagen-polyphenols-loaded silica composites were placed on two types of solid media (nutrient agar and yeast extract peptone agar) and incubated for 14 days at 37 °C.

### 2.10. Characterization Methods

The texture parameters of the functionalized mesoporous carriers were computed from the nitrogen adsorption/desorption isotherms acquired at 77 K on a Quantachrome Autosorb iQ_2_ gas sorption analyzer (Quantachrome Instruments, Boynton Beach, FL, USA). Pore size distribution and average pore diameter were calculated with Non-Local Density Functional Theory (NLDFT), specific surface area was determined in the range of relative pressure, *p*/*p*_0_, 0.02–0.25 with Brunauer–Emmett–Teller (BET) method, and total pore volume was assessed at *p*/*p*_0_ = 0.99.

Structural evaluation of MSN carriers was carried out by small-angle X-ray diffraction (XRD) on a Rigaku MiniFlex II diffractometer (Rigaku Corporation, Tokyo, Japan) using Cu-Kα radiation, in the range of 1.2–6 degrees (scanning speed 0.5 deg/min).

The chemical composition of the collagen-polyphenol-loaded silica composites was assessed by FTIR spectroscopy using the KBr technique. The FTIR spectra were recorded between 4000 and 400 cm^−1^_,_ with 64 scans, on a Bruker Tensor 27 spectrometer (Bruker Corporation Optik GmbH, Bremen, Germany).

The content of functional groups on the MSN carriers and common sage polyphenolic extract content was evaluated by thermogravimetric analysis (TGA, Netzsch STA 2500 Regulus, Selb, Germany) between 30 °C and 800 °C, heating rate 10 °C/min. All TGA measurements were performed under synthetic air.

The thermal stability of collagen and its composites was evaluated by differential scanning calorimetry on a Mettler Toledo (Greifensee, Switzerland) DSC 823e calorimeter operated at an N_2_ flow of 80 mL/min and a heating rate of 5 °C/min between 20 °C and 200 °C.

The morphology of MSN and composites was evaluated through scanning electron microscopy (SEM) and transmission electron microscopy (TEM). SEM images were collected on a Tescan Vega 3 LMH microscope (Brno, Czech Republic) coupled with an energy dispersive X-ray (EDX) detector, and TEM micrographs were recorded on a TECNAI F30 G2 S-TWIN microscope (Hillsboro, Oregon, USA) with a maximum accelerating voltage of 300 kV with a field emission electron gun.

## 3. Results

Prior to development of collagen-polyphenols-loaded silica composites, all components were thoroughly characterized. The structural and textural characteristics of MSN were evaluated by small-angle XRD, nitrogen adsorption/desorption isotherms, SEM, TEM, TGA, and FTIR.

### 3.1. MSN Carriers Characterization

The first step in design of novel composites consisted of preparation of MSN, which were used as a carrier for the polyphenolic extract. Pristine MSN were prepared by the sol–gel method, and, after template agent removal, they were further used to obtain functionalized MSN. Structural, textural, and morphological characteristics for pristine and functionalized silica carriers were evaluated.

**Figure 1:**
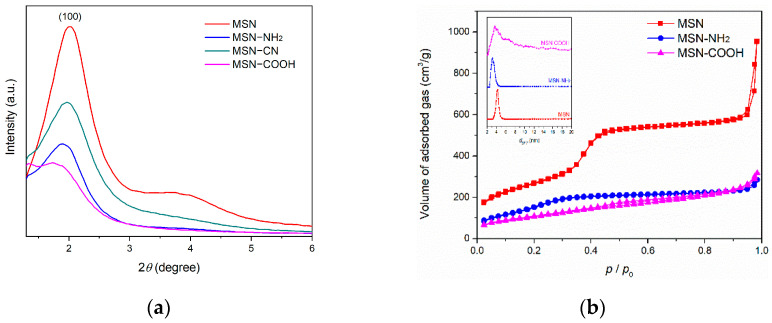
Small-angle XRD patterns (**a**) and nitrogen adsorption/desorption isotherms with pore size distribution in the inset (**b**) of MSN carriers.

The small-angle XRD pattern of the pristine MSN (Figure 1a, red curve) indicated a pseudohexagonal disordered pore array displaying an intense broad diffraction peak corresponding to the Bragg reflection and a small shoulder that can be attributed to the overlapped reflections (110) and (200), which are characteristic of the hexagonal pore array. Introduction of functional groups reduces the intensity of the main diffraction peak, as shown for MSN–NH_2_ (blue curve) and MSN–CN (green curve). Hydrolysis of nitrile groups to form carboxylic acid moieties, in the presence of sulfuric acid, determined a more pronounced effect on the main diffraction peak reduction because of the hydrolysis reaction that produced a collapse of the mesostructure.

A decrease in textural parameter values (specific surface area, pore volume, and average pore diameter) of functionalized samples compared to pristine MSN is also noted (Table 1). The synthesis procedure yielded nanoparticles with high porosity, calculated from nitrogen adsorption/desorption isotherms (Figure 1b). Thus, pristine MSN have a large specific surface area of 977 m^2^/g and a total pore volume of 1.43 cm^3^/g, which enabled further modification of their surface with organic groups. Attachment of functional groups through post-synthesis method reduced the specific surface area and pore volume for MSN–NH_2_ and MSN–COOH carriers to 512 m^2^/g and 388 m^2^/g and 0.42 cm^3^/g and 0.47 cm^3^/g, respectively. The average pore diameter of the functionalized carriers also decreased compared to that of pristine MSN (4.25 nm) (Figure 1b-inset): 3.18 nm for the MSN–NH_2_ carrier and 3.66 nm for MSN–COOH. The pore size distribution curve (PSD) of MSN–COOH (magenta curve) is broader than that of pristine MSN and MSN–NH_2_ (Figure 1b-inset). Furthermore, the PSD curve does not show a unimodal distribution of the pore size, as with the other two silica carriers. This result is consistent with the XRD data for MSN–COOH, indicating an impact of acid hydrolysis on the mesophase structure.

**Figure 2:**
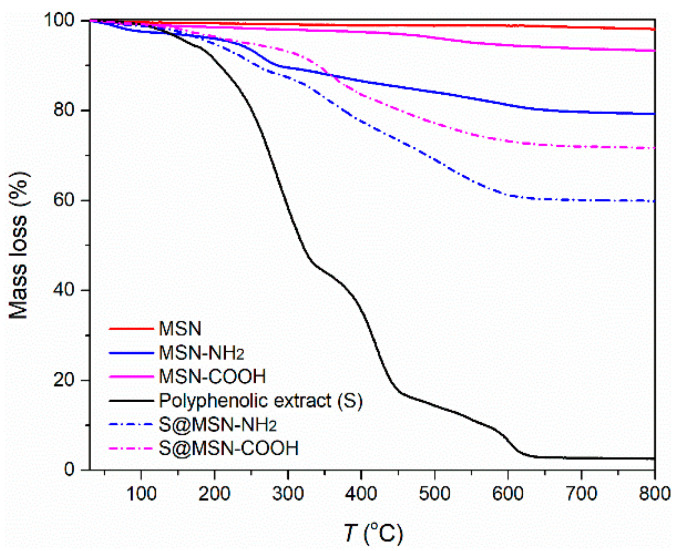
Thermogravimetric analysis of MSN carriers and polyphenolic-extract-loaded MSN.

The content of functional groups was determined from thermogravimetric analysis (Figure 2 and Table 1), disregarding the mass loss up to 100 °C, corresponding to the first endothermic event, which is assigned to physically adsorbed water on the silica surface of the mesoporous carriers. The mass loss between 100 °C and 650 °C was attributed to the thermal decomposition of the organic groups grafted on the surface of the silica. The content of organic functional groups (FG) on silica surface is listed in Table 1.

**Figure 3:**
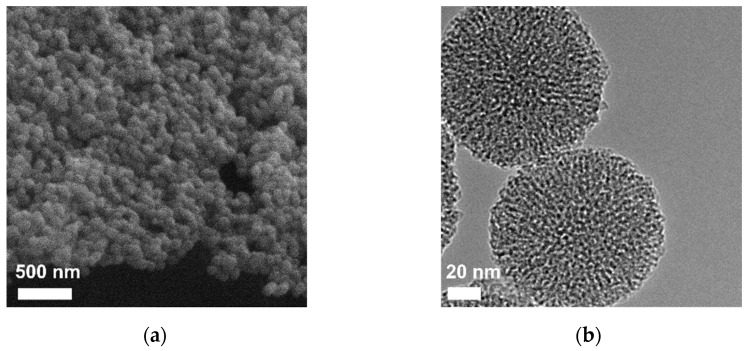
SEM image (**a**) and TEM image (**b**) of pristine mesoporous silica nanoparticles.

The morphology of MSN was assessed by SEM and TEM. The acquired SEM image (Figure 3a) shows that the nanoparticles have a spherical shape and uniform dimensions. The diameter of the nanoparticles was determined from SEM images by measuring at least 100 nanoparticles, and the average diameter was 105 ± 13 nm. The TEM image (Figure 3b) revealed more details of the nanostructure of the mesoporous carriers. The well-defined spherical nanoparticles present a mesopore array consisting of worm-like and cylindrical channels, consistent with the XRD results (Figure 1a).

**Figure 4:**
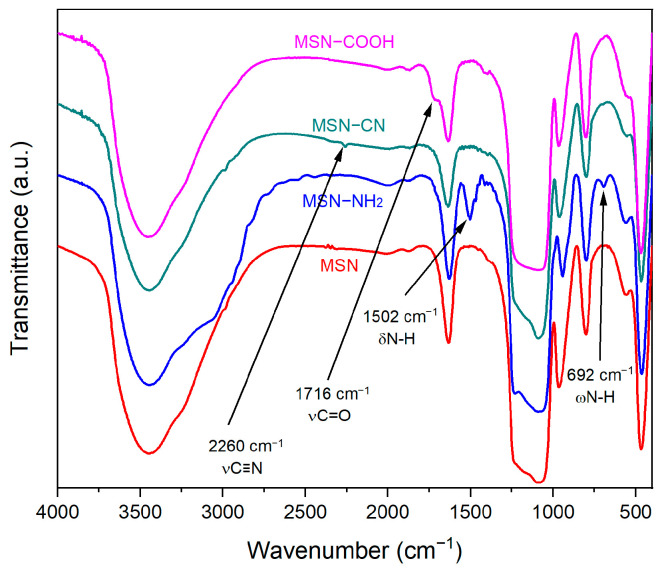
FTIR spectra of MSN supports.

Successful functionalization of MSN with organic moieties was demonstrated by FTIR spectroscopy (Figure 4). All carriers show the vibrations of physically adsorbed water molecules (1634 cm^−1^) and the vibration bands specific to the silica matrix: symmetric and asymmetrical stretching vibrations of the Si-O-Si bond (800 cm^−1^ and 1086 cm^−1^, respectively), Si-OH stretching vibrations (966 cm^−1^), Si-O symmetric bending vibration (468 cm^−1^), and the wide band corresponding to O-H stretching vibrations (2700–3800 cm^−1^). The characteristic vibrations of the functional groups grafted on the silica surface are observed: bending and wagging vibrations of the amine group (1502 cm^−1^ and 692 cm^−1^, respectively) and the carbonyl stretching vibration of the carboxylic acid group (1716 cm^−1^). The complete hydrolysis of the nitrile group to carboxylic moiety is confirmed by replacement of the nitrile band (2260 cm^−1^) identified in the MSN–CN spectrum with the C=O stretching vibration (1716 cm^−1^) in the MSN–COOH spectrum (Figure 4).

### 3.2. Polyphenols-Loaded MSN Composites Characterization

The source of the polyphenolic extract was the *Salvia officinalis* plant. The details of extract characteristics are provided in the Supplementary Materials. The chromatogram (HPLC-PDA, Shimadzu Nexera 2, Shimadzu Corporation, Kyoto, Japan) of the polyphenolic extract at 330 nm, the wavelength corresponding to the quantification of rosmarinic acid, is presented in Figure S1. Similar to previously reported *S. officinalis* extracts [34,40], the polyphenolic ethanolic extract obtained by ultrasound-assisted extraction at 40 °C was rich in rosmarinic acid, along with small quantities of caftaric acid, chlorogenic acid, caffeic acid, and rutin hydrate (Table S1). The presence of common sage polyphenolic extract into the mesopores of MSN carriers was evidenced in the FTIR spectra (Figure S2).

Total polyphenolic content (TPC) was determined by Folin–Ciocalteu method, and it was expressed as gallic acid equivalents (GAE) according to the method described previously [34]. The value of TPC for the polyphenolic extract was 163.78 ± 1.09 mg _GAE_/g _extract_.

The amount of polyphenolic extract loaded into the mesopores of the functionalized MSN was determined by thermogravimetric analysis (Figure 2). Thermal decomposition of the polyphenolic extract (black curve) occurred in the temperature range of 30–650 °C, after which the mass reached a plateau corresponding to a 2.84% residual mass consisting of inorganic compounds present in the extract. The results indicated 23.9% extract content for S@MSN–COOH and 26.6 % for S@MSN–NH_2_.

**Figure 5:**
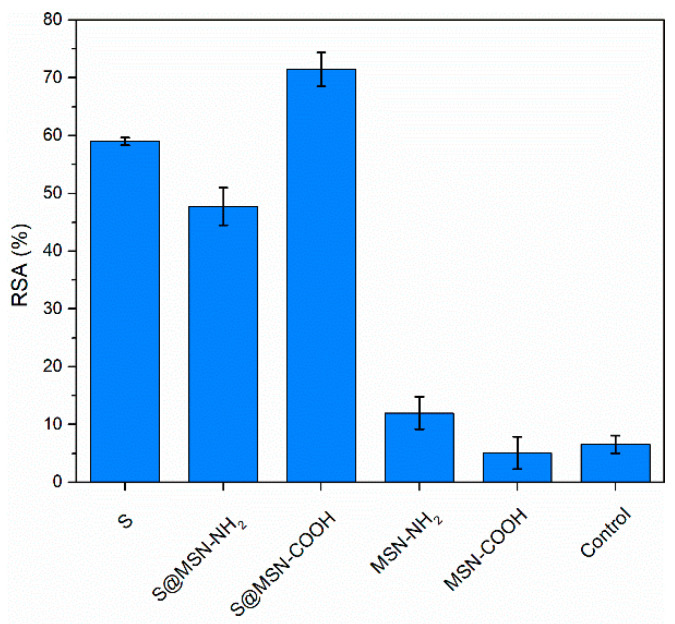
Radical scavenger activity of polyphenols-loaded MSN by the ABTS assay.

Radical scavenger activity (RSA) of *Salvia officinalis* extract was evaluated against ABTS˙^+^ radical, and polyphenolic extract RSA was 294 ± 3 mg _TE_/g _extract_. Among our previously reported extracts obtained from *S. officinalis* [34,40], the one used for developing collagen-polyphenols-loaded silica composites had the highest RSA value quantified through ABTS assay. The same method was applied for RSA evaluation of extract-loaded MSN. Mesoporous carriers alone were also tested to assess their contribution to RSA. The results obtained for the polyphenols-loaded MSN were compared with the ones for the same amount of mesoporous carrier and free extract, respectively, in the extract-loaded MSN (Figure 5).

Antibacterial activity of the common sage extract in comparison to that of the embedded extracts was assessed (Table 2). Stronger microbial growth inhibition was observed for the encapsulated extract, lower concentrations being necessary to interfere with the bacterial multiplication, the lowest MIC (3.9 mg/mL) being observed for S@MSN–COOH on *S. aureus*, while the lowest MBC obtained for the same sample on the same bacterial strain was 7.81 mg/mL. Among the bacterial strains, *S. aureus* showed the highest susceptibility to the tested extract.

**Figure 6:**
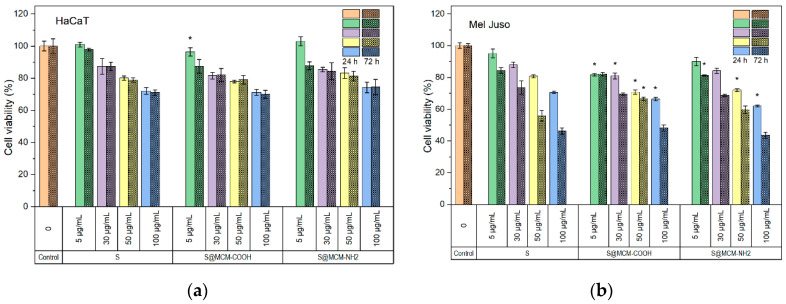
Cytocompatibility results of embedded and free extract on human keratinocyte normal cells (**a**) and antiproliferative activity on human melanoma cells (**b**). All results are expressed as mean values ± standard deviation (n = 3). * *p* < 0.05 compared to free extract, S (Student’s *t*-test).

Cytocompatibility and antiproliferative activity of free and embedded polyphenolic extract was evaluated by MTT assay after 24 h and 72 h incubation. The tested extract is cytocompatible, whether it is free or embedded into functionalized MSN, with cell viability values greater than 70% for normal human keratinocytes for both time points tested (Figure 6a). After 72 h of treatment, the percentage of cell viability in the group treated with *Salvia-officinalis*-embedded extract was comparable to that registered in the control group, the free *S. officinalis* group. The percentages of viable cells varied from 100% to 71%. All tested concentrations, including the highest 100 µg/mL, proved not to dramatically interfere with cellular proliferation.

The ability of *Salvia officinalis* extract encapsulated in mesoporous silica matrices to inhibit the cell proliferation process of Mel-Juso cancer cells was investigated. The common sage-extract-loaded functionalized MSN were tested on skin carcinoma cell line in order to extend the possible applications of developed composites for cancer-suffering people for a synergistic effect: anticancer and healing of skin-wounds. Based on MTT results, S@MSN–NH_2_ statistically significantly decreased (*p* < 0.05) the number of viable cells (62.09 ± 0.72%) at a concentration of 100 µg/mL at 24 h post-treatment (Figure 6b). Cell proliferation was also decreased in the case of S@MSN–COOH at the same tested concentration (66.36 ± 1.14%). The trend was maintained after 72 h of treatment and had an even higher impact on the ability of cells to proliferate, beginning with 50 µg/mL for the free extract. In terms of embedded extract, the number of viable cells has already been significantly reduced from 30 µg/mL for both S@MSN–COOH and S@MSN–NH_2_, with percentages of viable cells in the range of 69.37 ± 0.86% and 68.63 ± 0.75%, respectively.

**Figure 7:**
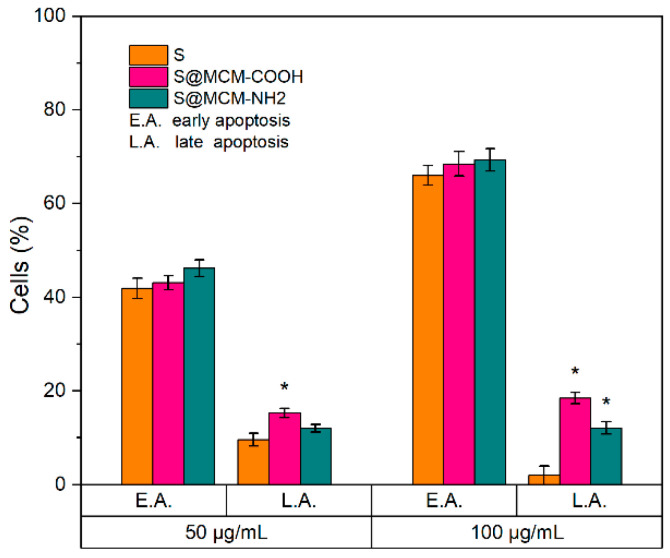
Flow cytometry analysis of cultured Mel-Juso cells’ mortality by apoptosis after exposure to free and embedded polyphenolic extract at 50 µg/mL and 100 µg/mL concentration for 72 h. Results are expressed as mean ± standard deviation (n = 3). * *p* < 0.05 compared to free extract, S (Student’s *t*-test).

The proportion of apoptotic cells with increasing sample dose of treatment added to the cultured Mel-Juso cells is shown in Figure 7. An increased proportion of apoptotic cells was detected compared to the control, 41.9%, 43.1%, and 46.2% when cultured Mel-Juso cells were incubated with samples S, S@MSN–COOH, and S@MSN–NH_2_, respectively, at concentrations of 50 µg/mL. At a concentration of 100 µg/mL, there was a progressive increase in percentages of apoptotic cell populations, with values of 66.0%, 68.5%, and 69.3% for samples S, S@MSN–COOH, and S@MSN–NH_2_, respectively. A statistically significant increasing proportion (*p* < 0.05) of apoptotic cells were identified in the late apoptosis stage for the encapsulated extract at 100 µg/mL concentration.

### 3.3. Collagen Porous Scaffold Composites Characterization

**Figure 8:**
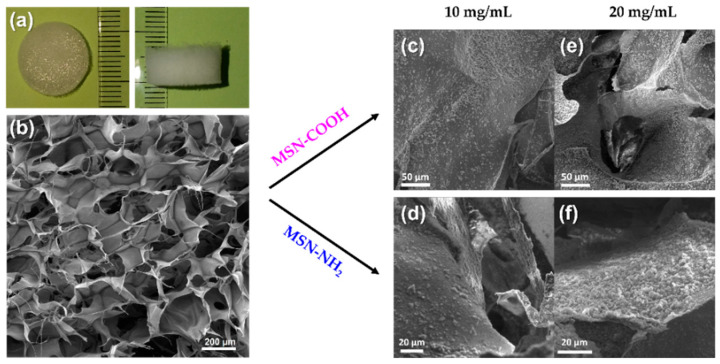
Photos of collagen porous scaffolds (**a**) and SEM images of collagen porous scaffolds (**b**); collagen–MSN composites prepared using 10 mg/mL (**c**,**d**) and 20 mg/mL (**e**,**f**) concentration of MSN.

Round-shaped collagen porous scaffolds were obtained after lyophilization with 15 mm diameter and 7 mm thickness (Figure 8a). The morphology of the collagen porous scaffold was assessed by SEM. Pristine porous scaffold exhibited a multilayered structure formed by sheets, with interconnected pores with diameters ranging from 0.05 to 0.6 mm (Figure 8b). Functionalized MSN were introduced into the collagen porous scaffold prior to lyophilization in various proportions to assess the structural features of the resulting collagen–MSN composites. The samples were denoted C@MSN-x (x = NH_2_ or COOH). Thus, the two types of functionalized MSN were added to the collagen suspension in various concentrations (10 or 20 mg MSN/mL collagen solution). At a higher concentration, MSN started to aggregate on collagen sheets, causing collapse of the scaffold structure under the weight of MSN (Figure 8e,f). At a concentration of 10 mg/mL, both types of MSN showed good integration into the collagen porous structure, being very well dispersed into the collagen scaffold (Figure 8c,d). The distribution of MSN in the collagen porous scaffold was examined by SEM coupled with energy dispersive X-ray analysis (Figure S3). The image demonstrated the MSN uniform distribution into the scaffold.

**Figure 9:**
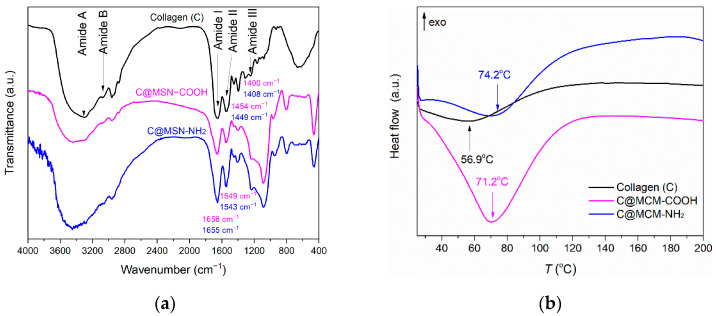
FTIR spectra (**a**) and DSC analysis (**b**) of collagen−MSN composites.

FTIR spectra of proteins have specific spectral regions [41] where vibrations of the amide bonds can be identified. Therefore, the FTIR spectra of the collagen porous scaffolds (Figure 9a) showed amide I vibration (1651 cm^−1^) corresponding to C=O stretching vibration, with contributions from C-N stretching and N-H bending vibrations; amide II (1542 cm^−1^) assigned to C-N stretching band combined with N-H in-plane bending vibration; amide III (1244 cm^−1^) belonged to N-H bending vibration coupled with C-N stretching vibration; amide A (3300 cm^−1^) and amide B (3074 cm^−1^) ascribed to N-H stretching vibration and asymmetrical stretching of CH_2_. Aside from the specific amide bands, other vibrations of the skeletal structure of collagen were identified: CH_3_ bending modes in skeletal proteins (1396 cm^−1^) and O-H bending vibration of COOH terminal groups that could overlap the C-H bending bands (1449 cm^−1^). In collagen–MSN composites, slight shifts in the bands of the collagen scaffold were noticed and displayed in Figure 9a.

The thermal stability of collagen–MSN composites in comparison with that of collagen scaffold was assessed. DSC analyses of the samples (Figure 9b) showed an endothermic event at 56.9 °C for the collagen porous scaffold, 71.2 °C for C@MSN–COOH, and 74.2 °C for C@MSN–NH_2_. The DSC results showed an enhanced denaturation temperature of collagen–MSN composites compared to that of pristine collagen porous scaffold.

**Figure 10:**
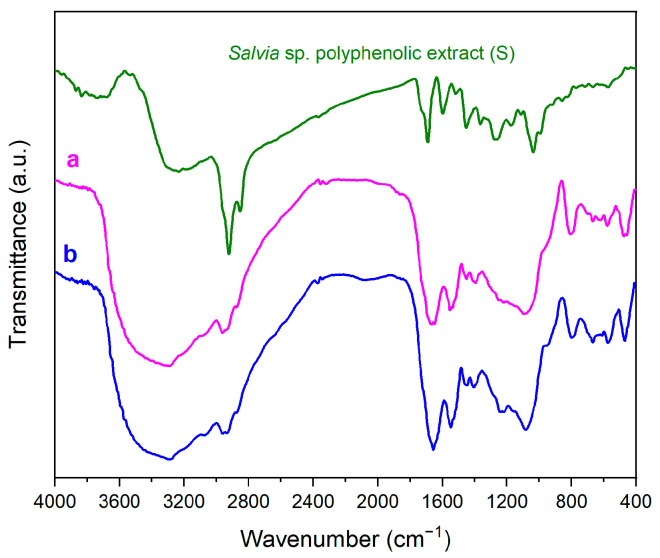
FTIR spectra of collagen−polyphenols-loaded MSN composites: collagen−S@MSN−COOH (a) and collagen−S@MSN−NH_2_ (b).

After complete characterization of the collagen porous scaffold and establishing the parameters regarding the amount of MSN that could improve the properties of the porous scaffold, collagen-polyphenols-loaded MSN were prepared and characterized. FTIR spectra showed intense amide I and amide II of the collagen porous scaffold (Figure 10), and more intense bands in the 1350–1475 cm^−1^ wavenumber range due to C-H bending vibrations that overlap bands of all three components of the composites. Moreover, in the range of 500–700 cm^−1^, bands of C-H out-of-plane bending vibrations are distinguished.

**Figure 11:**
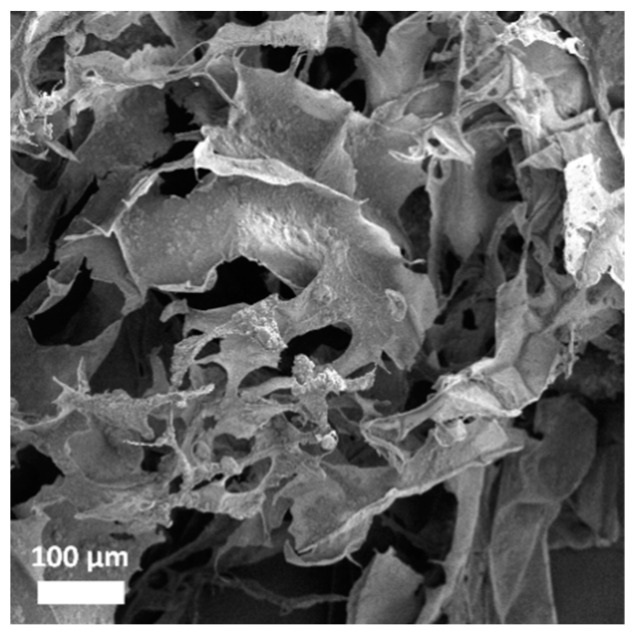
Microstructure of the collagen−polyphenols−loaded MSN−NH_2_ composite.

The morphology of both types of collagen-polyphenols-loaded silica composites was evaluated by SEM (Figure 11). The *S. officinalis*-extract-loaded MSN were homogenously distributed into collagen sheets, although the functionalized MSN were slightly more agglomerated after extract loading in their mesopores.

Sterility testing of the collagen porous scaffolds and their analogue composites with polyphenols-loaded-silica revealed that, after 14 days, no yeasts or molds were developed on nutrient agar or yeast-extract peptone culture medium.

## 4. Discussion

The developed collagen-polyphenols-loaded silica composites combine synthetic and natural components to obtain advanced materials. A polyphenolic extract was incorporated into synthetic mesoporous inorganic carriers and then included in a natural polymer matrix, a collagen porous scaffold.

MSN were prepared through sol–gel method using TEOS as silica precursor and CTAC as templating agent. The hydrolysis and condensation reactions of the silica precursor were catalyzed by TEA, which also assisted in achieving better particle size control. PEG is mostly used for coating of silica nanoparticles surface for biomedical applications because it shields the negative charge of the silica surface and helps in reducing the MSN interaction with biological molecules [42]. However, in the proposed synthesis procedure, PEG was not bound to the MSN surface; it was employed as an inhibitor of particles growth. The aim of this work was to obtain small silica nanoparticles that would enable good dispersion into the collagen porous matrix without influencing the microstructure of the porous scaffold. The MSN synthesis method yielded spherical nanoparticles with a diameter of around 100 nm and high porosity (*S_BET_* = 977 m^2^/g and *V* = 1.43 cm^3^/g).

Pristine MSN were further used for functionalized MSN synthesis. Moieties with acidic or basic nature able to form acid–base interactions and hydrogen bonding with the collagen matrix were grafted onto the MSN surface by a post-synthesis approach. Thus, 3-aminopropyl moieties were grafted on pristine MSN surface, creating MSN–NH_2_. For the acid moieties, propionitrile groups were grafted onto pristine MSN, which were then hydrolyzed in acidic medium to form propionic acid moieties (MSN–COOH).

Natural compounds, such as polyphenols, can be used as antibacterial and antifungal agents [18]. We selected *Salvia officinalis* (common sage) for the composites’ development as it was reported as a source of phytochemicals with biological activity. Literature data show that application of low-intensity ultrasound during the extraction process usually does not alter the vegetal material and its components, instead decreasing the extraction time, which is correlated with a higher yield in natural compounds compared to the conventional method [43]. For common sage extract, ultrasound-assisted extraction led to better recovery of natural compounds as a result of breaking down plant tissues into the solvent containing the vegetal material. The total polyphenol content (TPC) of the prepared common sage extract was determined using Folin–Ciocalteu reagent and expressed as gallic acid equivalents (163.78 mg _GAE_/g _extract_). The ultrasound-assisted extraction yielded a high TPC value, larger than the previously reported values for the conventional method (138.11 mg _GAE_/g _extract_) [40] or MW-assisted extraction (119.85 mg _GAE_/g _extract_) in absolute ethanol [34]. Furthermore, a high concentration of rosmarinic acid was obtained through this extraction method (25.56 mg/g _extract_), along with small quantities of caftaric acid, chlorogenic acid, and caffeic acid. Rosmarinic acid exhibits biological activities, such as antioxidant, anti-inflammatory, and antidiabetic, and also has antimicrobial properties [44].

Entrapment of polyphenolic compounds into mesopores of MSN has been shown to be beneficial in terms of maintaining the radical scavenger activity of an extract [37,45]. For this reason, this strategy was approached with the *S. officinalis* extract to ensure good stability over time. The results obtained from determination of RSA using ABTS assay indicated slightly better antioxidant activity of the embedded extract in MSN–COOH. For the S@MSN–NH_2_ sample, the RSA value is lower than that of the free extract (S), but there are some interferences because of the carrier that seems to interact with ABTS radical.

The antibacterial activity of polyphenols-loaded MSN was tested against two bacterial strains, *Staphylococcus aureus* and *Pseudomonas aeruginosa*, and compared to the one of the free extract. The two bacterial strains are the most common pathogens representative of Gram-positive and Gram-negative bacteria classes that are associated with skin infections as a result of injuries, i.e., burns [46,47]. The results illustrated better inhibitory and bactericidal activities for the polyphenols-loaded MSN compared to the free extract. Moreover, there are differences between the two types of bacteria: lower values of MIC and MBC were demonstrated for *Staphylococcus aureus*, most likely due to the different composition and structure of cell envelope of the bacterial strains.

To further address the aesthetic aspect in long-term healing of skin wounds, a collagen porous scaffold was considered for development of complex composites. Natural protein fibers such as collagen promote cellular regeneration, and, since they are naturally occurring compounds found in the epithelial tissue, they have good biocompatibility [5]. The marine-source collagen used in these composites comes from *Rapana venosa*, which is a sustainable source of collagen.

The collagen structure consists mainly of glycine, proline, and hydroxyproline, and the chains of the triple helix are connected through glycine bonds. The positions of amide I and II in the FTIR spectra have been shown to be related with the secondary structure of proteins [48]. Thus, the position of the amide I band at 1651 cm^−1^ indicated the α-helical structure of the collagen. Minor shifts (4–8 cm^−1^) of the main amide bands of collagen in the case of collagen–MSN composites are only due to the overlap of the vibrations characteristic of the functional groups of MSN with those of the amino acids in the protein. A disruption of the α-helix of the collagen structure would shift the position of the amide I band towards higher frequencies [49], but the position of amide I band of collagen–MSN composites did not reveal such a perturbation on the collagen secondary structure. Amide A and amide B superimposed the broad band of the silanol groups of MSN supports. In the FTIR spectrum of the collagen–MSN–NH_2_ composite, the N-H stretching vibration band of MSN–NH_2_ also overlapped the amide A and B band, and, therefore, it is difficult to assign its position.

DSC data were recorded to assess the thermal behavior of the collagen porous scaffolds. The endothermic event identified in the 30–100 °C range is attributed to the collagen denaturation temperature. The pristine porous scaffold showed a denaturation temperature of 56.9 °C, while the presence of functionalized MSN led to a higher denaturation temperature, above 70 °C, for both collagen-functionalized MSN composites. Denaturation temperature is strongly associated with proteins structure. The endothermic event is due to transformation of the collagen secondary structure from a triple-helix to a random coil structure [50]. This transformation occurs as result of elimination of water molecules strongly bound through hydrogen bonds, which stabilize the triple-helix structure. The DSC results indicated enhanced thermal stability of the collagen–MSN porous scaffold compared to the pristine one, probably due to the electrostatic interactions between the functional groups of MSN and the amino acids from the collagen structure.

The collagen-polyphenols-loaded silica composites retained the same microstructure as the pristine collagen porous scaffold, a structure with multiple layers and interconnected pores, that showed good distribution of polyphenols-loaded MSN on the sheets of the collagen matrix.

It is very well known that natural products stored in open air are always susceptible to fungal contamination; thus, molds and/or yeasts can easily grow. Prevention of such contamination is a critical aspect considering that the envisioned role of the composites is prevention of bacterial infection in wound healing applications. Both the collagen porous scaffold and its composites containing polyphenols-loaded silica exhibited good sterility, which demonstrated high stability against development of fungi for long-term storage of the composite materials.

A large variety of phytocompounds have demonstrated in vitro encouraging activity against oxidative and proliferative processes [51]. The antitumor potential of common sage extracts has been evaluated in various cancerous cell lines, as well as animal models. Pro-apoptotic and antiproliferative effects have been reported on cell lines of breast (MCF-7) [52], cervix adeno carcinoma (HeLa) [53], colorectal cancer (HCT-116, HCT15, CO115, HT-29), and melanoma (A375, M14, A2058, B16) [35].

Analysis of apoptosis showed that, for the S@MSN–NH_2_ sample, a relatively higher percentage of apoptotic cell population was obtained, these results being correlated with that obtained from antiproliferative analysis by MTT assay. To conclude, the encapsulated common sage extract did inhibit tumoral cell growth after 72 h, with an impact on apoptosis induction, and had no effect on the viability of normal keratinocytes cells.

## 5. Conclusions

This work reported for the first-time development of collagen-polyphenols-loaded silica composites. The newly designed composites comprised polyphenolic extract with good antibacterial properties, which was embedded into mesopores of functionalized mesoporous silica carriers. Polyphenolic-extract-loaded MSN showed good preservation of radical scavenging activity and enhanced antibacterial activity on both Gram-negative and Gram-positive bacterial strains. The final collagen-polyphenols-loaded silica composites exhibited excellent stability against fungus development. Furthermore, introduction of MSN carriers into the collagen porous scaffold increased the denaturation temperature of the protein, improving the thermal stability of the composites.

The challenges of designing tissue engineering composites consist of reaching an equilibrium between the physicochemical characteristics and biological properties of the composites. The design of collagen-polyphenols-loaded silica composites represents a new approach to use of natural compounds for wound dressings. The advantages of using such composites are: (i) use of renewable sources for their design (polyphenolic extract prepared from *S. officinalis* plant and collagen scaffold obtained from an invasive snail, *R. venosa*), (ii) exploring new antibacterial agents as an alternative to typical antibiotics, (iii) high loading capacity of polyphenols and their protection against degradation provided by mesoporous silica carriers, (iv) support in cell regeneration offered by the collagen matrix, (v) good biocompatibility, and (vi) antiproliferative effect on skin carcinoma cells.

These composites that address multiple issues in one application represent a promising perspective on tissue engineering, but further knowledge in this research area is necessary to develop new biocompatible matrices with advanced properties.

## Figures and Tables

**Table 1:** Textural parameters of MSN carriers.

Sample	*S*_BET_ m^2^/g	*d*_DFT_ nm	*V* cm^3^/g	*V*_d < 10 nm_ cm^3^/g	SiO_2_:FG
MSN	977	4.25	1.43	0.78	-
MSN–NH_2_	512	3.18	0.42	0.32	1:0.22
MSN–COOH	388	3.66	0.47	0.29	1:0.05

*S*_BET_—specific surface area; *d*_DFT_—average pore diameter; *V*—total pore volume; *V*_d < 10 nm_—pore volume for pores with diameter less than 10 nm; FG—functional groups.

**Table 2:** Antibacterial activity of free common sage extract and embedded in functionalized MSN.

Sample	MIC (mg/mL)		MBC (mg/mL)	
	*S. aureus*	*P. aeruginosa*	*S. aureus*	*P. aeruginosa*
S	15.62	31.25	31.25	62.0
S@MSN–NH_2_	7.81	15.62	15.62	31.25
S@MSN–COOH	3.90	15.62	7.81	31.25

MIC—minimum inhibitory concentration; MBC—minimum bactericidal concentration.

## Data Availability

Not applicable.

## Supplementary Materials

The following supporting information can be downloaded at: https://www.mdpi.com/article/10.3390/pharmaceutics15020312/s1, Figure S1. HPLC chromatogram of *Salvia officinalis* polyphenolic extract; Table S1. Composition of *S. officinalis* polyphenolic extract; Figure S2. FTIR spectra of polyphenols-loaded MSN; Figure S3. SEM-EDX image of the collagen–MSN–NH_2_ composite.
