# Supplementary material for: Novel Collagen-Polyphenols-Loaded Silica Composites for Topical Application
Source: Pharmaceutics. 2023 Jan 17;15(2):312. doi: 10.3390/pharmaceutics15020312 (PMC9962153; doi:10.3390/pharmaceutics15020312)
Supplement: Supplementary file 1 [file pharmaceutics-15-00312-s001.zip › pharmaceutics-2132091-supplementary.pdf]

Novel Collagen-Polyphenols-Loaded Silica Composites for Topical Application

Mihaela Deaconu [1,2], Ana-Maria Prelipcean [3,*], Ana-Maria Brezoiu [2], Raul-Augustin Mitran [4], Gabriela Isopencu [2], Cristian Matei [2], Daniela Berger [2,*]

[1] CAMPUS Research Institute, University "Politehnica" of Bucharest, 313 Splaiul Independentei, 060042 Bucharest, Romania
[2] Faculty of Chemical Engineering and Biotechnologies, University "Politehnica" of Bucharest, 1-7 Polizu Street, 011061 Bucharest, Romania
[3] National Institute of R&D for Biological Sciences, 296 Splaiul Independetei, 060031 Bucharest, Romania
[4] "Ilie Murgulescu" Institute of Physical Chemistry, Romanian Academy, 202 Splaiul Independentei, 060021 Bucharest, Romania
* Correspondence: annastanciuc@gmail.com (A.-M.P.); daniela.berger@upb.ro (D.B.)

1. *Characterization of polyphenolic extract obtained from Salvia officinalis*

The composition of the polyphenolic extract obtained from *Salvia officinalis* by ultrasound-assisted extraction in ethanol, at 40 °C was evaluated through high-performance liquid chromatography with photodiode array detector (HPLC-PDA). according to the previously reported method [26].

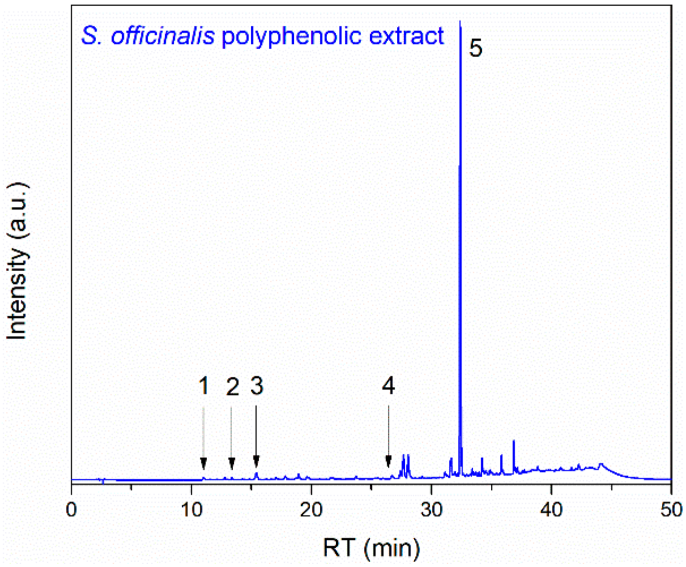

Figure S1. HPLC chromatogram of *Salvia officinalis* polyphenolic extract

Table S1. Composition of *S. officinalis* polyphenolic extract

No.	Compound	*RT* min	*c* mg compound / g extract
1	Caftaric acid	11.09 ± 0.07	0.551 ± 0.022
2	Chlorogenic acid	13.06 ± 0.03	0.197 ± 0.045
3	Caffeic acid	15.58 ± 0.06	0.409 ± 0.008
4	Rutin hydrate	26.44 ± 0.04	0.632 ± 0.006
5	Rosmarinic acid	32.27 ± 0.01	25.564 ± 0.180

RT – retention time; *c* – concentration.

2. *Characterization of polyphenolic extract-loaded carriers*

FTIR spectra of polyphenols-loaded MSN (Figure S2) exhibit the vibrations of the functionalized silica carriers, which have been already described in section 3.1., together with those of the polyphenols present in the *S. officinalis* extract. Thus, the most intense bands are the sharp bands at 2920 cm^{-1} and 2850 cm^{-1}, which are ascribed to the stretching vibrations of the C-H bonds, followed by the vibration of the C=O bonds around 1700 cm^{-1}. The intensity of the other bands belonging to the extract are attenuated after the adsorption of the extract into the mesopores of the carriers, most of them overlap the silica bonds.

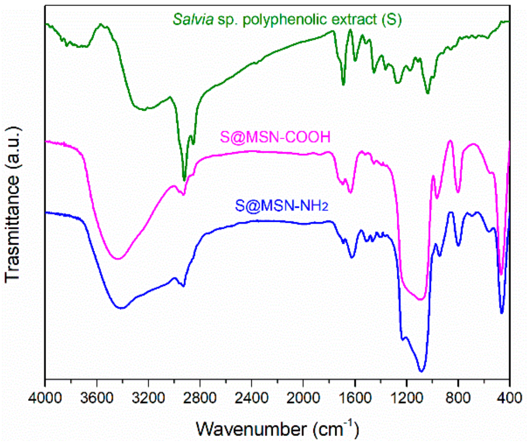

Figure S2. FTIR spectra of polyphenols-loaded MSN

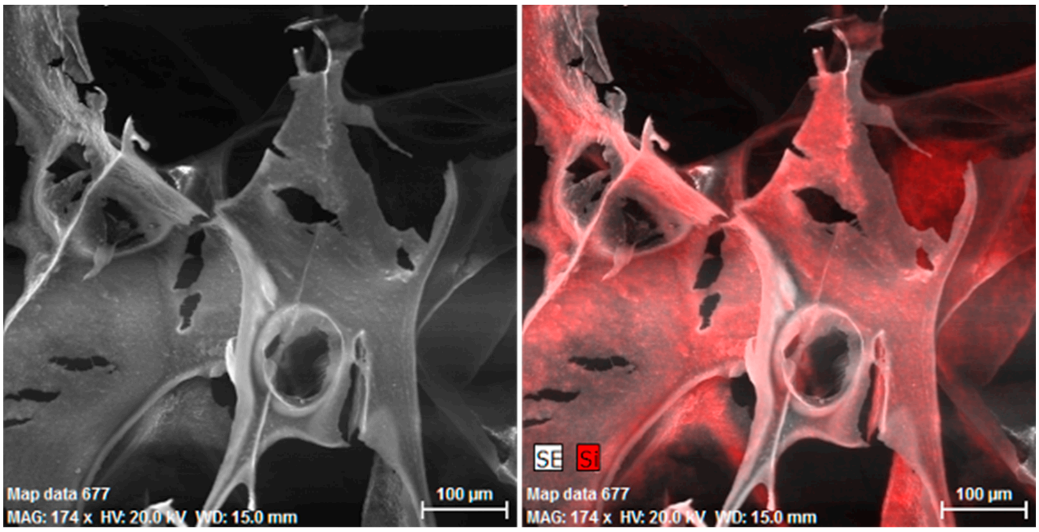

Figure S3. SEM-EDX image of the collagen-MSN-NH_2-composite
